# In vitro detection of canine anti-human antibodies following intratumoral injection of the hu14.18-IL2 immunocytokine in spontaneous canine melanoma

**DOI:** 10.1371/journal.pone.0330200

**Published:** 2025-08-19

**Authors:** Andrew T. Kosharek, Cindy L. Zuleger, William S. Glass, Jens Eickhoff, Paul M. Sondel, David M. Vail, Mark R. Albertini

**Affiliations:** 1 University of Wisconsin School of Medicine and Public Health, Madison, Wisconsin, United States of America; 2 Department of Medicine, University of Wisconsin School of Medicine and Public Health, Madison, Wisconsin, United States of America; 3 Carbone Cancer Center, University of Wisconsin, Madison, Wisconsin, United States of America; 4 University of Wisconsin-Madison, Madison, Wisconsin, United States of America; 5 Department of Biostatistics & Medical Informatics, University of Wisconsin School of Medicine and Public Health, Madison, Wisconsin, United States of America; 6 Department of Human Oncology, University of Wisconsin School of Medicine and Public Health, Madison, Wisconsin, United States of America; 7 Department of Pediatrics, University of Wisconsin School of Medicine and Public Health, Madison, Wisconsin, United States of America; 8 Department of Medical Sciences, University of Wisconsin School of Veterinary Medicine, Madison, Wisconsin, United States of America; 9 Department of Dermatology, University of Wisconsin School of Medicine and Public Health, Madison, Wisconsin, United States of America; 10 The Medical Service, William S. Middleton Memorial Veterans Hospital, Madison, Wisconsin, United States of America; Colorado State University, UNITED STATES OF AMERICA

## Abstract

**Background:**

Canine and human malignant melanoma are naturally occurring cancers with many similarities, making the dog an important parallel patient population to study both diseases. However, development of canine anti-human antibodies (CAHA) needs to be considered when evaluating humanized biotherapeutics in dogs.

**Objectives:**

Characterize CAHA in sera from dogs with spontaneous melanoma receiving radiotherapy and intratumoral immunocytokine (IT-IC) with humanized 14.18-IL2.

**Methods:**

Serum samples were obtained pre-treatment and at several post-treatment times from 12 dogs with locally advanced or metastatic melanoma treated with radiotherapy to the primary site and regional lymph nodes (when clinically involved) followed by IT-IC of humanized 14.18-IL2. Two CAHA assays were developed. A sandwich enzyme-linked immunosorbent assay (ELISA) was developed to detect antibodies against the humanized IgG component of hu14.18-IL2. A flow cytometry assay was developed to determine the ability of CAHA to inhibit binding of a mouse anti-GD2 monoclonal antibody to its target.

**Results:**

Post-treatment sera from 7 of 12 dogs developed CAHA levels over pre-treatment that were identified by ELISA as significant increases at Day 30 and/or Day 60. Day 10, Day 30, and Day 60 post-treatment sera from 10 of 12 dogs significantly inhibited the binding of anti-GD2 monoclonal antibody to its target compared to pre-treatment. Significant binding inhibition was also detected in 2 of 12 dogs after local RT but before IT-IC (Day 1). Normal canine sera did not mediate binding inhibition.

**Conclusions:**

This study advances CAHA detection strategies and reports the kinetics of CAHA following IT-IC in dogs with spontaneous melanoma.

## Introduction

Companion (pet) dogs with spontaneous tumors provide unique and clinically relevant insights into human tumor biology, therapy, and drug development [1,2]. Dogs develop spontaneous tumors, similar to humans [3]. This contrasts with many laboratory mouse models where tumors are experimentally induced [4]. Moreover, the molecular and genetic characteristics of many canine cancers resemble those of human cancers [5]. Spontaneous canine melanoma is a serious disease with a prognosis that depends on clinical stage [6–8]. While murine models are critical for initial *in vivo* studies, there is a disconnect between the number of anti-cancer therapeutics that work in mice versus in humans. In contrast, many human clinical trials have benefitted from the translational dog model [9]. Malignant melanoma is the most common canine oral malignancy and is associated with high rates of metastasis similar to advanced stage melanoma in people. Canine oral melanomas are highly malignant tumors, with metastasis occurring via lymphatics or blood vessels to regional lymph nodes, lungs, liver, brain, and kidney [2,10,11]. Oral malignant melanoma in dogs has similar biologic behavior to that of cutaneous melanoma in people and most closely resembles the acral lentiginous form [12]. Canine analogs of human melanoma antigens have been detected in canine melanomas [13]. Moreover, similar to human melanoma, analyses of canine melanomas have revealed a broad spectrum of somatic mutations [14,15]. Thus, the mutational landscape of canine melanoma recapitulates that of human melanoma, further strengthening the validity of testing novel immunotherapies that target unique tumor neoantigens.

While no model perfectly recapitulates human cancer, the companion dog melanoma patient population holds a distinctive position in oncology research and has received attention from the scientific community as being somewhat parallel to human melanoma patients, particularly for evaluation of immunotherapy studies [16–20]. In that regard, development of antidrug antibodies (ADAs) against immunotherapeutic monoclonal antibodies (mAbs) is well documented in human patients, and the presence of ADAs can impact the safety and efficacy of immunotherapy [21–25]. Moreover, the immune response is highly species-specific and relatively few immunotherapeutic mAbs are caninized, i.e., canine speciated [26]. Therefore, it will be critical to monitor ADAs in the setting of canine immunotherapy, particularly when the immunotherapeutic is a mAb of a species ‘foreign’ to the dog (e.g., a humanized mAb).

Canine and human melanoma share important clinical and biological features, including aggressive local invasion, spontaneous onset, histopathologic subtypes and metastatic potential. Molecular similarities have been described including comparisons between RAS and PI3K-AKT pathways [27,28]. These parallels support utility of the canine model for evaluating immunological responses to therapies, including biologics.

**Fig 1:**
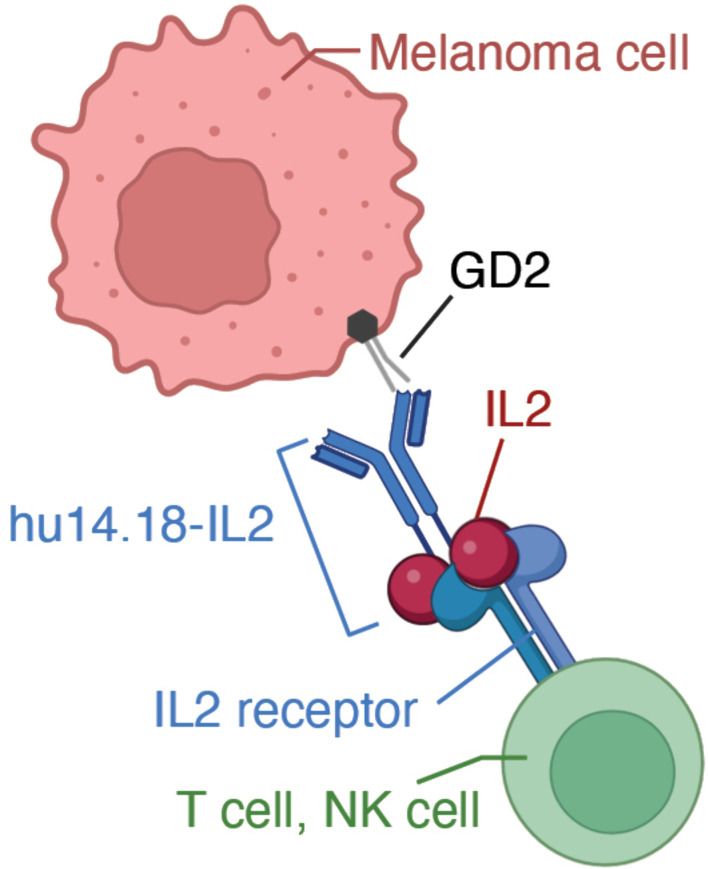
Immunocytokine hu14.18-Il2. The mAb hu14.18 is specific for GD2 expressed on tumor cells and is linked to two molecules of IL2 at the Fc region which serve to attract immune effector cells to the tumor microenvironment. Figure adapted from [47]. Created in BioRender. Zuleger, C. (2025) https://BioRender.com/yq3u26o.

The immunocytokine (IC) hu14.18-IL2 links a humanized anti-GD2 monoclonal antibody (mAb), hu14.18, that retains only the complementarity-determining region of the original mouse antibody, covalently to two molecules of IL-2 at the Fc region [29,30] (Fig 1). The antigen binding region of hu14.18 mAb recognizes GD2, a membrane-bound disialoganglioside found on tumors of neuroectodermal origin (including melanoma, neuroblastoma, and certain sarcomas) with relatively scarcity on normal tissues (mostly cerebellum and peripheral nerves) [29]. The linked IL2 molecules provide a milieu of increased IL2 concentration and function to chemoattract immune cells, e.g., T cells and NK cells to the tumor microenvironment [31]. The GD2 disialoganglioside (GD2) is expressed not only in murine melanoma, but importantly, also in both human and spontaneous canine melanoma [32–35]. In this way, antibodies that target GD2 are akin to tumor-specific reagents. Hu14.18-IL2, and the chimeric murine-human ch14.18-IL2, have been studied *in vitro* and in preclinical mouse models with GD2-expressing tumors [36–38]. Clinical trials evaluating intravenous (IV) delivery of hu14.18-IL2 IC in patients with melanoma or neuroblastoma have demonstrated immune activation and had manageable safety profiles [39–43]. Although hu14.18-IL2 is a humanized mAb, some patients still developed antibodies to the therapeutic IC [40,44]. In these patients, antibodies were detected to both the idiotype, i.e., the antigen end, and to the Fc-IL2 end of the IC [40,44]. A substantial fraction of patients develop a detectible anti-IC antibody response, and in some patients the response is so strong that IC subsequently administered is undetectable in the blood directly after administration – effectively an in vivo “neutralization” of the IC [40,44]. Neutralizing human anti-human antibody (HAHA) or human anti-chimeric antibody (HACA) have also been reported for some patients treated with humanized Hu14.18K322A or chimeric hu14.18 mAb [45,46].

The immunogenicity of the humanized hu14.18-IL2 is potentially a greater concern when administering this molecule to a “foreign” species, e.g., to a mouse or dog, as ADAs could interfere with the pharmacokinetics of this therapy, potentially reducing its efficacy [48]. When huKS1/4-IL2, a humanized antibody directed against the human epithelial cell adhesion molecule, was administered intravenously to mice with adenocarcinoma, the mice mounted significant ADAs in the form of mouse anti-human antibodies (MAHAs) resulting in rapid clearance of additional doses of the therapeutic [49]. Delivering hu14.18-IL2 intratumorally in mouse models also induced a MAHA response, similar to what was seen following IV-IC therapy [50]. In addition, ADAs in dogs have been observed in other studies, including one report following subcutaneous administration of a therapeutic IC [51–53]. However, the development of canine anti-human antibodies (CAHAs) following intratumoral immunotherapy with an IC has not been reported.

Here we present CAHA assay development and CAHA monitoring of companion dogs with spontaneous melanoma enrolled in a clinical trial of local RT plus intratumoral hu14.18-IL2. An in-house ELISA was developed and used to monitor CAHA against the IgG portion of the IC. Previous studies have demonstrated the ability of ELISA to detect ADAs against antibody-based biopharmaceuticals [44,54–57]. Our ELISA is customizable, cost-effective, and uses common laboratory reagents and equipment. Using a GD2-specific mAb as a surrogate for the IC, we also examined if CAHA interferes with the IC’s ability to bind GD2 in a cell-based flow cytometry assay. Details about safety, clinical activity, and tumor biopsy analyses from this trial, as well as another trial, are separately reported [35,58].

## Materials and methods

### Trial eligibility and enrollment

Trial eligibility and inclusion criteria have been previously described [35]. Client-owned (pet) dogs weighing ≥5 kg with a histologically confirmed malignant melanoma diagnosis with a readily palpable and accessible tumor site of at least 2.0 cm allowing for IT-IC administration were eligible for study. Melanoma staging used the TNM classification of tumors in domestic animals staging system [59]. Both treatment naïve dogs and dogs with surgically recurrent tumors were allowed, provided the recurrence met the minimum size criteria. Prior radiation therapy or immunotherapy were exclusion criteria and a minimum 2-week washout from previous chemotherapy was required. Concurrent corticosteroid use was prohibited. All dogs were required to have a pretreatment constitutional clinical sign status of 0 or 1 (normal or mild lethargy over baseline; diminished activity from pre-disease level but able to function as an acceptable pet) according to VCOG-CTCAE v2 criteria at study entry [60]. Dogs determined to have any significant co-morbid illness were excluded. Only dogs for whom owners signed the informed consent to be screened and treated were tracked in this study. All cases deemed ‘not eligible’ from first contact or physical exam to proceed with screening were not tracked. As such, only 1 dog did not continue after screening and this dog entered another clinical trial. Details are provided in the study flowchart (S1 Fig).

### Study design

**Fig 2:**
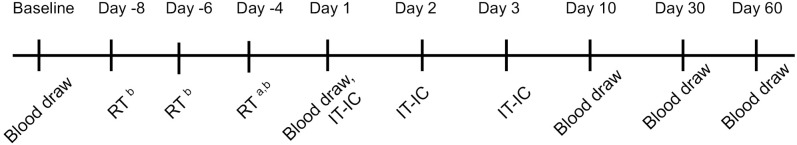
Treatment Schema. All dogs receive radiation therapy (RT) to the local site either in 1x 8 Gy (Arm A) or 3x 8 Gy fractions over 1 week (Arm **B)**. RT was delivered on Day −4 (Arm A) or on Days −8, −6, and −4 (Arm **B)**. Intratumoral (IT) injection of IC (IT-IC) is administered on three consecutive days starting five days after completion of RT. All days were allowed ± 1-2 days leeway for flexible scheduling around holidays, subject vacations, and clinic scheduling. ^a^Arm A, a single 8 Gy fraction; ^b^Arm B, three 8 Gy fractions delivered on a Monday, Wednesday and Friday schedule.

Client-owned companion dogs with locally advanced or metastatic melanoma were randomized (6 dogs per Arm) to receive either a single 8 Gy fraction (Arm A) or three 8 Gy fractions delivered over 1 week (Arm B) to the primary site and regional lymph nodes (when clinically involved) with the single or last fraction 5 days prior to IT-IC at 12 mg/m^2^ on 3 consecutive days. Lymph nodes were sampled and included in the RT plan only if clinically involved (i.e., positive by cytology) and only positive nodes were included. Prior to treatment, a baseline physical examination with tumor measurements was performed, blood was collected for clinical assessments and for banking of serum, plasma, and peripheral blood mononuclear cells (PBMC) for subsequent *in vitro* analysis. Blood was collected into BD Vacutainer® serum tubes (BD Biosciences Cat# 367820), allowed to clot and processed according to manufacturer’s instructions. Sera was aliquoted to microcentrifuge tubes (~1 ml per tube) and frozen at −80°C. Clinical tumor staging included aspirate of regional lymph nodes as well as thoracic radiographs and/or thoracic/abdominal CT as clinically indicated. External beam radiation therapy (RT) was delivered by image-guided intensity modulated radiation using helical tomotherapy (TomoTherapy HiArt Treatment System, Accuray Inc., Sunnyvale, CA) as previously described [35]. The hu14.18-IL2 was provided as lyophilized vials (4 mg/vial; each ml prior to lyophilization contained 4 mg/ml hu14.18-IL2, 2% sucrose, 80mM L-arginine, 10mM citric acid, 0.2% polysorbate 20, pH 5.5) and was reconstituted with either 0.5 ml or 0.25 ml 0.9% (w/v) NaCl for injection at a concentration of either 8 mg/ml or 16 mg/ml. The hu14.18-IL2 was delivered intratumorally, not intravenously, by inserting the needle into the target site along the longest diameter of the lesion and the target was then infused with hu14.18-IL2 as the needle was withdrawn to disperse along the length of the longest diameter. The total volume for injection (0.25 cc to 1.0 cc) was case-dependent on the size of the target tumor at the time of injection and was administered using multiple needle re-directions into the target lesion as previously described [35]. Injections were performed on 3 consecutive days to the same target area each day. Blood samples were obtained at pre-treatment and various times post-treatment. Etomidate (1–2 mg/kg, IV) and/or Propofol (4–5 mg/kg IV) were used to induce anesthesia in dogs receiving RT. Isoflurane (1.2–1.3%, inhalation) and/or Sevoflurane (2.4%, inhalation) were used to maintain anesthesia and the gas percentages were adjusted depending on patient’s clinical response to anesthesia under guidance of the clinician. The duration of anesthesia was approximately 30 minutes. Butorphanol (0.2–0.4 mg/kg IM or IV) and/or midazolam (0.2 mg/kg IV) were used to sedate dogs during IT-IC administration, the duration of which was approximately 45 minutes. Pulse, respiratory and heart rates were monitored throughout anesthesia and at least every 15 minutes after until the dog was ambulatory. While sedated, pulse and respiratory rate were monitored at least every 15 minutes until the dog was ambulatory. Lidocaine was administered at the site of the IT-IC administration for pain (0.5–0.75 ml 2% Lidocaine, intradermal). Following IT-IC administration, pain was alleviated with analgesics for 3 days or longer if the clinician or owner perceived that the dog was experiencing continued discomfort. Analgesics included either Carprofen (2.2 mg/kg, orally, 2x daily), Deracoxib (3–4 mg/kg, orally, 1x daily), or Meloxicam (0.1–0.2 mg/kg, orally, 1x daily); and/or Tramadol (1–5 mg/kg, orally, every 8 hours). Physical examination and patient history were assessed at each clinic visit. Dogs were monitored by the dog’s owner and clinician for animal health and behavior, as well as the development of adverse events (AE). AEs were graded and attributed according to VCOG-CTCAE v2 [60]. There were no AEs observed greater than Grade 2 and the number of clinical responses were too small for conducting meaningful analyses evaluating the correlations between CAHA responses and clinical responses. The attending clinician prescribed appropriate treatment and/or supportive care if an AE was observed. All efforts were made to minimize suffering. The study endpoint was the time the dog developed progressive disease as determined by physical examination and/or thoracic radiographs. Owners could remove a dog from the study for any reason, or the attending clinician could remove a dog from the study if it was determined there were severe AEs not manageable with supportive care. Euthanasia was not a study endpoint nor was it part of this study and was only performed at the request of a dog’s owner. If requested, the American Veterinary Medical Association guidelines were followed [61]. The treatment schema and blood collection times are shown in Fig 2.

### IACUC approvals

The authors confirm that the appropriate ethical review committee approval has been received. All procedures and treatments performed on client-owned companion dogs were approved by the Institutional Animal Care and Use Committees of the University of Wisconsin-Madison School of Veterinary Medicine (Approval V006037) and by the Animal Care Committee at the William S. Middleton Memorial Veterans Hospital. Written informed consent was obtained from all companion dog caregivers prior to entry into this trial. Protocol treatments were administered at the University of Wisconsin Veterinary Care (UWVC) hospital.

### Canine anti-human ELISA

A standard protocol was adapted to develop an ELISA to detect canine antibodies against human IgG [62]. Nunc MaxiSorp™ Flat-Bottom 96-well plates (Thermo Scientific Inc. Cat# 44-2404-21) were coated with ChromPure Human IgG (Jackson ImmunoResearch Labs Cat# 009-000-003, RRID:AB_2337043) (10 μg/mL, 50 μL/well in ELISA Coating Buffer (Bio-Rad Laboratories, Inc. Cat# BUF030C)), which served as the “capture” agent, and incubated overnight at 4°C. The coating solution was removed, and the wells washed three times (3x) with distilled water (DI). The plate was blocked with Neptune™ Block (ImmunoChemistry Technologies Cat# 64) (200 μL/well) for 1 hour at 25°C followed by washing as above. All available sera for each dog were batch analyzed on the same day in quadruplicate and on the same plate. Canine serum was diluted 1:20 in PBS and added to wells in quadruplicate at 50 μL/well and incubated for 1 hour at 25°C. A murine anti-human IgG (Abcam Cat# ab436, RRID:AB_955947) mAb was used as a positive control, as we are not aware of any commercially available canine anti-human IgG that could be used as a positive control. The mAb was diluted 1:1,000 in PBS and added to wells in quadruplicate at 50 μL/well, equivalent to ~255 ng/well, and incubated for 1 hour at 25°C. PBS was added to wells as a negative control instead of mAb or sera. Positive and negative controls, murine anti-human IgG mAb and PBS, respectively, were assayed on each plate concurrently with sera from dogs in this study. Wells were washed as above. Rabbit anti-mouse IgG (H + L) antibody, affinity purified against human serum proteins conjugated to horseradish peroxidase (HRP) (Jackson ImmunoResearch Labs Cat# 315-035-045, RRID:AB_2340066) was diluted 1:40,000 in PBS and added to wells at 75 μL/well as the secondary (i.e., “detection”) antibody and the plates were incubated for 30 minutes (min.) at 25°C. Wells were washed as above. The plate was developed with 1-Step™ Ultra TMB-ELISA solution (Thermo Scientific Inc. Cat# 34028) (75 μL/well) and incubated for 15 min. at 25°C. The reaction was stopped by addition of TMB stop solution (Sera Care Cat# 5150−0020) (75 μL/well), and the absorbance at 450nm read on a SpectraMax M3 Micro-Plate reader.

### Cell culture

The GD2 + M14 human melanoma cell line (RRID:CVCL_1395) was authenticated by the University of Wisconsin Translational Research Initiatives in Pathology (TRIP) Laboratory (S2 Fig) and cultured in RPMI-1640 (Corning Cat# 15–040-CV) supplemented with 10% fetal bovine serum (GeminiBio, BenchMark™ Cat# 100–106), 25 mM HEPES (Corning Cat# 25–060-CI), 2 mM L-glutamine (Corning Cat# 25–005-CI), 1 mM sodium pyruvate (Corning Cat# 25–000-CI), and 1X non-essential amino acids (Corning Cat# 15–025-CI)). M14 was cultured in Falcon 75 cm^2^ tissue culture flasks (Corning Cat# 353136) and maintained at 37°C in a humidified 5% CO_2_ atmosphere. The flask surface was rinsed with PBS, incubated in the presence of 1 ml 1x trypsin (Corning Cat# 25–053-CI) for 2–3 minutes, and the flask surface was rinsed with culture media to collect the cells. A GD2 expressing, human, melanoma cell line, kindly provided by Dr. Ralph Reisfeld of The Scripps Research Institute ~35 years ago, and was provided to him by Dr. Don Morton of UCLA, was recently proven by STR to be the M14 melanoma line that was originally derived at UCLA (S2 Fig).

### Detection of GD2 binding inhibition by CAHA using flow cytometry

All available sera for a dog were batch analyzed on the same day in triplicate. Mouse anti-GD2 mAb 14G2a-PE (BioLegend Cat# 357303, RRID:AB_2561884) was diluted 1:320 in 1% FBS/PBS flow cytometry wash buffer (WB) and 100 μl added to microcentrifuge tubes. Baseline, Day 1, Day 10, Day 30, Day 60 sera from dogs in this study (2.5 μl) was added to diluted anti-GD2 antibody.. Whole (non-immune) dog sera (2.5 μl or 20 μl) (Antibodies Incorporated Cat# 54–490, RRID:AB_2797201), and hereafter referred to as ‘Healthy’, was added to diluted anti-GD2 antibody. The total volume of all samples was adjusted to 120 μl with WB such that the sera from this study were diluted 1:48 and healthy dog sera was diluted 1:6 or 1:48. A sample without canine sera was used as a staining control. Controls were assayed concurrently with sera samples from dogs in this study. The light-protected mixture was incubated for 30 min. at 4°C. The serum-14G2a mixture was added to GD2 + M14 cells (0.1 x 10^6^ M14 cells per tube) aliquoted to flow cytometry tubes (Corning Cat# 352008) and incubated for 30 min.at 4°C, followed by 2x washes with WB. The viability of M14 cells was > 95% by trypan blue exclusion prior to use in the assay. M14 cells were fixed with 300 μl Cytofix Fixation Buffer (BD Biosciences Cat# 554655) for 15 min. at 25°C, washed 1x with WB, resuspended in WB, and held at 4°C until analysis. Data were acquired on a BD LSR Fortessa, spurious events and doublets excluded, single cells were gated, and the Median Fluorescence Intensity (MFI) was determined with FlowJo^TM^ v10.10 Software (BD Life Sciences). The gating strategy is shown in S3 Fig. Binding inhibition was calculated as follows where X equals Day 1, Day 10, Day 30, or Day 60:


$$\%\text{ Binding\textbackslash{} Inhibition}=100\times \frac{\text{Baseline\textbackslash{} MFI}-\text{Day\textbackslash{} X\textbackslash{} MFI}}{\text{Baseline\textbackslash{} MFI}}\;$$ (1)

### Statistical analysis and considerations

A sample size of 6 dogs per arm (total = 12 dogs) was planned for this study. This sample size was selected as it was adequate to detect anticipated moderate to large effect sizes with sufficient power when comparing candidate biomarker levels between arms. Specifically, a sample size of 6 dogs per arm provides between 49–94% power at the one-sided 0.05 significance level to detect anticipated effect sizes ranging between 1.0–2.0 standard deviation units in candidate biomarker levels. Due to the exploratory nature of this study, there was no blinding in the study and there were no multiple testing adjustments for evaluating multiple candidate biomarkers. Animal specific kinetic levels of CAHA following IT-IC were summarized in terms of means and standard errors. Profile plots were generated to examine changes over time. Changes from baseline to Day 1, 10, 30, and 60 were evaluated using a generalized linear model. Model assumptions were validated by examining residual and normal probabilities plots. Dunnett’s test was used to control the type I error (<0.05) when conducting multiple comparisons for changes from baseline to Day 1, 10, 30, and 60. Pearson’s correlation analyses were conducted to evaluate the associations between median fluorescence intensity levels obtained from flow cytometric vs. ELISA assays within each Arm and for both Arms combined. All reported P values are two-sided and P < 0.05 was used to define statistical significance. Statistical analyses were conducted using SAS software (SAS Institute, RRID:SCR_008567), version 9.4. Plots were generated using R software version 4.5.0 with graphical packages [63–65]. The computer codes which were used to conduct the analyses described in the manuscript were written in SAS and R. The codes will be shared on reasonable request to the corresponding author.

## Results

### Companion dog characteristics

Twelve dogs met eligibility criteria for the trial and were enrolled and randomized into one of the two treatment groups from May 2020 to December 2021 [35]. Companion dog breed, tumor characteristics, and randomization results are summarized in Table 1. All 12 dogs completed the treatment phase of the protocol, and none experienced significant or unexpected adverse events [35]. The sex, age and weight of the enrolled dogs were previously reported and are presented in Table 1 [35]. Day 60 serum was not available for two dogs (ITIC-06 and ITIC-12) (Table 1). The time between peripheral blood sampling at Baseline and at Day 1 is presented in Table 2.

**Table 1:** Characteristics and status of dogs receiving 12 mg/m^2^ IC and randomized to RT Arm A or RT Arm B.

Patient ID	Breed	Sex	Age (yr)	Wt (kg)	Target Tumor Site & Stage at Diagnosis^a^	RT Arm^b^	Sera available from all tmpts?	Recurrent Disease^c^
**ITIC-04**	Labrador Retriever	SF	10	38.9	Left Maxilla/Pulmonary nodule (T2N0M1)	A	Yes	No
**ITIC-05**	Burnese Mountain	NM	7	43.4	Right hindlimb subcutaneous (T2N2M0)	B	Yes	Yes
**ITIC-06**	Min. Poodle mix	NM	10	6.6	Right Mandible (T2N1M0)	A	No^d^	Yes
**ITIC-07**	Aust. Cattle mix	NM	13	31.2	Left Maxilla (T2N0M0)	B	Yes	No
**ITIC-08**	Beagle mix	NM	11	15.8	Soft palate (T2N1M0)	B	Yes	No
**ITIC-09**	Great Dane	SF	10	51.7	Left Maxilla (T2N1M1)	A	Yes	Yes
**ITIC-10**	German Shepherd	NM	9	26.3	Right Maxilla (T2N0M0)	A	Yes	No
**ITIC-11**	Mix Breed	NM	11	26.5	Right Mandible (T2N0M1)	B	Yes	No
**ITIC-12**	Pom. mix	SF	11	9.0	Left Maxilla (T3N1M0)	A	No^d^	Yes
**ITIC-13**	Mix Breed	NM	10	7.0	Cutaneous (TxN1M1)^e^	A	Yes	Yes
**ITIC-14**	Collie mix	SF	11	25.0	Left Maxilla (T2N1M0)	B	Yes	Yes
**ITIC-15**	Mix Breed	NM	9	36.1	Lingual (T2N0M0)	B	Yes	No

IC, immunocytokine; RT, radiation therapy; ID, identification study number; yr, year; Wt, weight; tmpts, timepoints; ITIC, intratumoral immunocytokine; SF, spayed female; NM, neutered male; Min., miniature; Aust., Australian; Pom., Pomeranian

^a^Melanoma staging used the TNM classification of tumors in domestic animals staging system [59].

^b^Arm A, a single 8 Gy fraction to the primary tumor followed 5 days later with hu14.18-IL2 IC (12 mg/m^2^ for 3 consecutive days); Arm B, three 8 Gy fractions delivered on a Monday, Wednesday and Friday schedule followed 5 days later with hu14.18-IL2 IC (12 mg/m^2^ for 3 consecutive days).

^c^All dogs with therapeutic melanoma management before study entry were managed by surgical excision except for ITIC-14 which also received hypofractionated RT (4 weekly fractions of 8 Gy) 5 months prior to a subsequent recurrence and entry into this clinical trial.

^d^Serum not available from Day 60.

^e^The primary tumor had been excised prior to this trial. Therefore, the target tumor for RT and ITIC was the effaced lymph node.

**Table 2:** Number of days between baseline and day 1.

Patient ID	# Days^a^
**ITIC-04**	12
**ITIC-05**	16
**ITIC-06**	9
**ITIC-07**	9
**ITIC-08**	9
**ITIC-09**	12
**ITIC-10**	7
**ITIC-11**	9
**ITIC-12**	5
**ITIC-13**	5
**ITIC-14**	9
**ITIC-15**	8

^a^The number of intervening days between the Baseline and Day 1 blood samples.

### Detection of CAHAs by ELISA

We developed an in-house sandwich ELISA to detect antibodies in canine sera specific to human IgG — the antibody portion of hu14.18 is humanized IgG. For this assay to work, a secondary antibody specific for, or cross-reactive to, canine immunoglobulin is required. Additionally, to avoid the need for multiple secondary reagents, reactivity to mouse immunoglobulin, the species in which the positive control is raised, was preferred. We first tested our potential secondary reagents by optimizing their ability to generate a strong signal when detecting mouse anti-human IgG mAb as the positive control (in place of the canine serum). Our initial “checkerboard” trials tested various concentrations of human IgG coating antigen, mouse anti-human IgG mAb as the analyte, and rabbit anti-dog HRP secondary antibody. As expected, the measured absorbance was dependent on the concentration of the rabbit anti-dog HRP secondary, i.e., absorbance decreased as concentration of the secondary decreased. In contrast, the absorbance was not dependent on the concentration of either the coating human IgG or the mouse anti-human IgG mAb (S4 Fig). To address these issues, we first troubleshot reagents and/or steps, e.g., testing different blocking buffers, including 5% normal rabbit sera, as well as non-mammalian protein buffers, increasing the blocking time, and testing different wash buffers. These changes did not resolve the high background issue, which was also observed in negative control wells, i.e., in wells incubated with PBS instead of the mouse anti-human IgG mAb.

These data suggested that the rabbit anti-dog HRP secondary may bind to the human IgG coating on the plate. Immunoglobulins from different species share similar protein structures [62]. Therefore, antibodies against one species are likely to cross-react with several other species — a property which, as discussed below, was leveraged to optimize the assay. We adsorbed the rabbit anti-dog secondary antibody with excess human IgG; however, recognition of human IgG by this adsorbed reagent was only slightly minimized (S5 Fig). We are not aware of a commercially available anti-dog secondary antibody adsorbed to human serum proteins. Using the cross-reactivity of different species’ immunoglobulins, we next tested a commercially available rabbit anti-mouse IgG secondary antibody adsorbed to ensure minimal cross-reactivity with human serum proteins. The human adsorbed rabbit anti-mouse IgG did not bind to plate-bound human IgG while the cross-reactivity to the positive control mouse IgG was maintained (S6 Fig). This one reagent then permitted the use of a single secondary antibody to effectively detect both CAHA and mouse-anti-human antibody (MAHA).

Primary-only and secondary-only control wells were not included in the final ELISA protocol. Nonspecific background was assessed during the assay development using PBS-only wells. Elevated background was observed and attributed to cross-reactivity between the rabbit anti-dog IgG HRP secondary antibody and the plate-bound human IgG (S4 Fig). Using this rabbit anti-dog HRP secondary reagent the absorbance did not correlate with the concentration of the positive control mouse IgG mAb (compare absorbance across rows in S4 Fig). Further, a substantial signal was detected in the absence of the positive control mouse IgG mAb (note absorbances in column 6 in S4 Fig). In-house cross-adsorption of the rabbit-anti-dog HRP with human IgG did not sufficiently reduce the background (S5 Fig). Additionally, the finding of strong signal in wells without the positive control mAb (note absorbances in columns 3, 6, and 9 in S5 Fig) suggested the rabbit anti-dog IgG may bind directly to the human IgG coated wells. When the rabbit anti-dog IgG HRP was replaced with the commercial rabbit anti-mouse IgG HRP cross-adsorbed against human serum proteins (RRID:AB_2340066) the non-specific background was reduced while the positive control murine anti-human IgG signal was preserved (S6 Fig).

**Fig 3:**
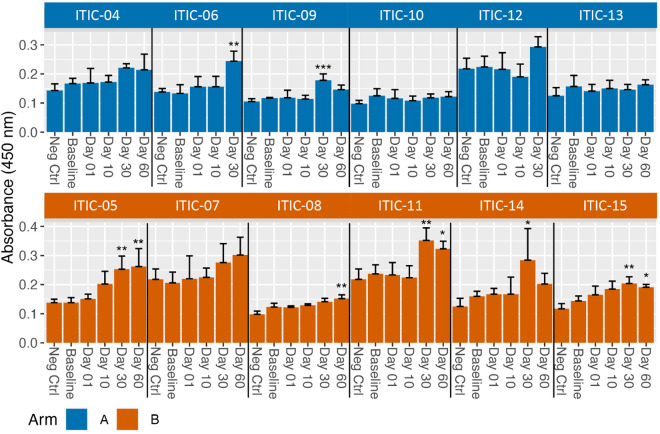
CAHA is induced in some dogs receiving IT-IC injections. Mean CAHA levels, measured as **O.**D. values by ELISA, from sera assayed in quadruplicate collected at Baseline (before radiation), Day 1 (after radiation, but prior to IT-IC), and Days 10, 30 and 60 (after IT-IC) with standard deviation error bars are shown. Day 60 timepoint was not available for two dogs, ITIC-06 and ITIC-12, in Arm **A.** Increases in absorbance from Baseline to Day 1, Day 10, Day 30, or Day 60 for each dog were analyzed and significance indicated as * p < 0.05; ** p < 0.01; *** p < 0.001.

Post-treatment sera from seven of twelve dogs tested positive for anti-human IgG antibodies determined via our in-house CAHA ELISA (Fig 3 summary data, S7 Fig replicate data). Pre-treatment sera were used as baseline and as an internal control for each dog’s post-treatment samples. Two of six and five of six dogs in Arm A and Arm B, respectively, demonstrated significant increases in anti-human IgG compared to the pre-treatment absorbance (Fig 3). The post-treatment anti-human IgG was significantly increased compared to pre-treatment anti-human IgG at Day 30 for six dogs and at Day 60 for four dogs. For Arm A, both samples reaching significance were from Day 30 (ITIC-06 and ITIC-09). The sera from ITIC-09 no longer had a significant increase in CAHA at Day 60, and no sera was available for Day 60 CAHA analysis from ITIC-06. For Arm B, anti-human IgG antibody levels in three dogs were elevated similarly at Day 30 and Day 60 (ITIC-05, ITIC-11, ITIC-15), one dog had an elevated level at Day 30 that declined by Day 60 (ITIC-14), and one had an elevated level that was only detected at Day 60 (ITIC-08). No dogs had detectable anti-human IgG antibodies following RT but before IT-IC (Day 1) or at Day 10 post-treatment. There were no substantial differences in anti-human IgG levels between negative control wells with PBS only and pre-treatment samples for the 12 dogs enrolled in this study.

### Sera from dogs treated with hu14.18-IL2 inhibit in vitro binding to GD2

We modified a flow cytometry assay to determine the potential for CAHA in canine sera to inhibit the binding of the IC to GD2 in a cellular model [50]. Sera from treated dogs is incubated with the anti-GD2 mAb clone 14G2a, and subsequently the mixture is used to stain M14 cells, a highly GD2 + human melanoma cell line. Clone 14G2a is an IgG2a-class switch variant of the mouse IgG3 mAb clone 14.18 and therefore has the same idiotype as that of the humanized immunocytokine hu14.18-IL2 [31]. Consequently, antibodies in dog sera against the idiotype of hu14.18 will also recognize and bind to the idiotype of 14G2a, effectively interfering with the ability of 14G2a to bind its GD2 target on M14 cells. The assay utilizes the detection of GD2 expression on M14 by 14G2a conjugated to fluorescent PE (14G2a-PE) as a flow cytometry readout. Thus, a canine serum only control (no 14G2a-PE) is not included because without the 14G2a-PE reagent, only background signal is detected which would result in falsely negative data. The assay does not determine if the dogs develop antibodies against the GD2 antigen itself. The fluorescence level of 14G2a-PE bound to M14 in the absence of sera is used as the reference control. If the fluorescence signal decreases in the presence of sera from hu14.18-IL2 treated dogs, CAHA recognized the 14G2a idiotype and effectively blocked its binding to M14. In contrast, little to no loss in fluorescence indicates that the serum antibodies did not block the binding of 14G2a to GD2. Importantly, as 14G2a is a mouse anti-human mAb, using the human melanoma M14 cell line as its target is appropriate [31].It is also possible that some CAHA might recognize other regions of the IC and these may not be detected by our assay.

In preliminary experiments, we assayed whole non-immune canine sera (obtained from Antibodies Incorporated RRID:AB_2797201 and hereafter referred to as ‘Healthy’) for components in dog serum that might interfere with the flow cytometry assay. Serum is a complex biologic that contains carbohydrates, proteins, and lipids that can interfere with the ability of antibodies to bind their targets. Healthy serum was spiked into PBS and incubated with the 14G2a anti-GD2 mAb before adding the mixture to M14 cells, as described above. Compared to M14 stained in the absence of Healthy sera, the addition of sera substantially inhibited 14G2a binding to GD2 in a concentration dependent manner. Substantial inhibition (~50.2%) was observed at 1:6 ratio of normal sera to PBS whereas at a 1:48 ratio inhibition was largely undetected (> 0.1%) (S8 Fig). As the observed interference of Healthy control serum on 14.G2a-PE binding to M14 was ameliorated at the 1:48 ratio, we used this ratio to test sera from the clinical trial. We included Heathy donor sera diluted 1:6 and 1:48 with PBS as positive and negative, respectively, controls in each assay. Each dog’s pre-treatment sample was used as its baseline.

**Fig 4:**
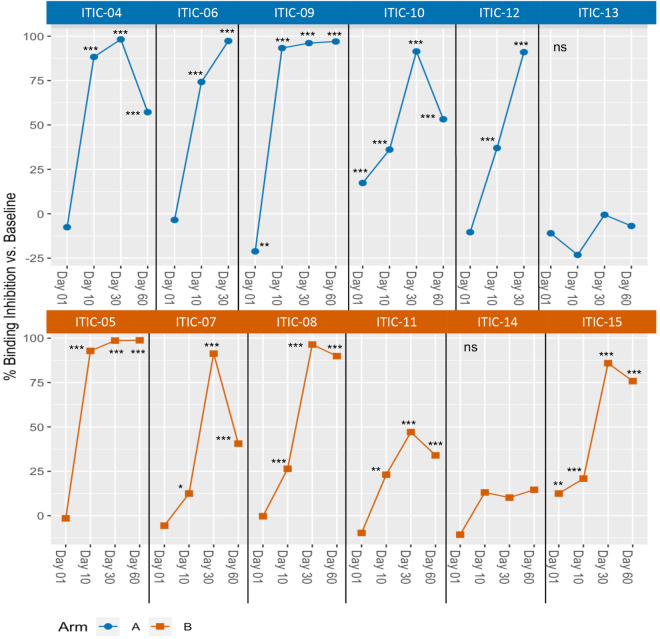
In vitro inhibition of binding of anti-GD2 antibody to GD2 target. The % binding inhibition at various timepoints compared to Baseline. Data shown are calculated from the mean of triplicates as described in Materials and Methods. Sera was collected at Baseline (before radiation), Day 1 (after radiation, but just prior to IT-IC), and Days 10, 30 and 60 (after IT-IC). Timepoints for each dog are presented and connected by a line. Day 60 timepoint was not available for two dogs, ITIC-06 and ITIC-12, in Arm **A.** The indicated significance is based on mean of triplicates at Baseline compared to post-treatment timepoints, * p < 0.05; ** p < 0.01; *** p < 0.001.

Varying levels of binding inhibition were observed in 10 of 12 dogs (all but ITIC-13 and ITIC-14) when canine sera were mixed with 14G2a mAb before the mixture was used to stain M14 cells (Table 3, Fig 4, and S9 Fig for selected data). The binding was significantly inhibited compared to pre-treatment in 10 of 12 dogs at Days 10 and 30 and in 8 of 8 dogs from whom the Day 60 sample was available (ITIC-06 and ITIC-12 did not have day 60 serum sample collected). GD2 binding was also significantly inhibited by Day 1 sera from 2 of 10 dogs, one dog in each Arm, (ITIC-10 and ITIC-15). Interestingly, the MFI fluorescence readout was increased (not decreased/inhibited) in the presence of Day 1 sera from ITIC-09 compared to pre-treatment, resulting in significantly lower binding inhibition (Fig 4 summary data, S9 Fig replicate data).A trend of such an increase in MFI (opposed to a decrease) was also noted at Day 1 compared to pre-treatment for 7 additional dogs, however these changes did not reach significance (Fig 4, S9 Fig, and Table 3). A positive binding inhibition value indicates that the Day 1 sera from ITIC-10 and ITIC-15 inhibited the binding of the 14G2a to the M14 cells. In contrast, a negative binding inhibition value indicates that the Day 1 sera increased, rather than decreased, the binding of the 14G2a to the M14 cells. The result of an increase, even slight, in binding in the presence of Day 1 sera compared to pre-treatment sera yields a binding inhibition value that is less than 0% (i.e., the negative binding inhibition value). These relatively small positive or negative binding inhibition values detected on Day 1 (prior to any IT-IC treatment, but after RT), which are significant only for 3 dogs (ITIC-09, ITIC-10, and ITIC-15), were not anticipated and are discussed below; for all 3 of these dogs, all 3 subsequent time points for each dog show higher and statistically significant binding inhibition. In contrast to the 10 dogs that did show significant binding inhibition, sera from the other 2 dogs (ITIC-13 and ITIC-14), did not inhibit GD2 binding at any timepoint (Fig 4 and Table 3). While the subject numbers in this study are too small to correlate CAHA responses to clinical responses or to AEs we provide the following context for the above-mentioned dogs. Briefly, ITIC-13 had advanced disease (Stage 4) when enrolled in the trial and did not respond well with stable disease at Day 30, progressive disease at Day 60, and progression-free survival (PFS) of 60 days. In contrast, ITIC-14 had Stage 3 disease when enrolled and had the best response of all dogs on study with a partial response at Day 30, complete response at Day 60, and PFS of 564 days. Neither of these two dogs had AEs greater than a Grade 1 nor were their presentations unusual. Further, we previously detailed the clinical responses and AEs of all dogs in this clinical trial [35]. Selected flow cytometry data are shown in S10 Fig.

**Table 3:** Binding Inhibition (%) of anti-GD2 mAb to GD2 target in the presence of canine serum.

Patient ID	RT Arm	Day 1	Day 10	Day 30	Day 60
**ITIC-04**	A	−7.6	88.3	98.2	57.2
**ITIC-05**	B	−1.5	92.8	96.6	98.8
**ITIC-06**	A	−3.5	74.2	97.3	n/a
**ITIC-07**	B	−5.6	12.5	91.3	40.6
**ITIC-08**	B	−0.3	26.4	96.4	89.9
**ITIC-09**	A	−21.2	93.3	96.1	97.0
**ITIC-10**	A	17.3	36.1	91.4	53.2
**ITIC-11**	B	−9.7	23.1	47.1	34.0
**ITIC-12**	A	−10.4	37.0	91.0	n/a
**ITIC-13**	A	−11.0	−23.2	−0.6	−6.9
**ITIC-14**	B	−10.7	13.1	10.2	14.6
**ITIC-15**	B	12.5	20.9	85.9	75.8

mAb, monoclonal antibody; RT, radiation therapy.

Fluorescently labelled anti-GD2 antibody was incubated with canine serum and then used to stain GD2 + M14 tumor cells. % Binding Inhibition of post-treatment samples compared to baseline samples was determined by flow cytometry as described in Materials and Methods.

### Comparison of CAHA Detection by ELISA and binding inhibition by *in vitro* flow cytometry assay

**Fig 5:**
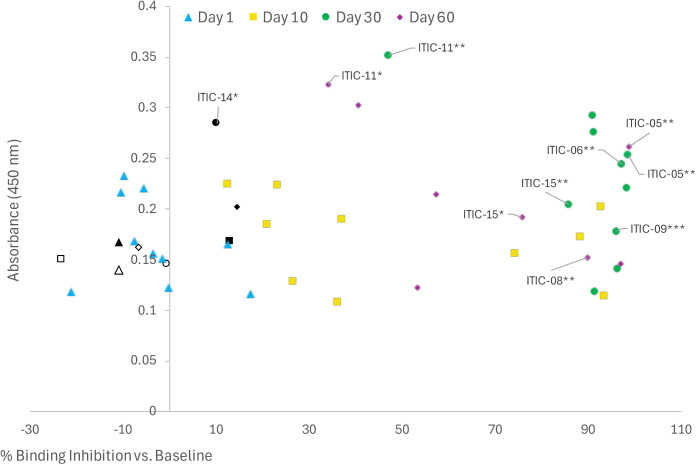
The relationship of CAHA and binding inhibition by *in vitro* flow cytometry assay. The % binding inhibition related to CAHA at various timepoints is shown. Timepoints for each dog are presented. Timepoints with significant CAHA responses are shown with significance indicated, these timepoints also had significant binding inhibition except for ITIC-14 Day 30. Sera from 10 of 12 dogs significantly inhibited binding of anti-GD2 antibody to GD2 target at Day 10, 30 and 60 with the exceptions of ITIC-13 (shown as open symbols) and ITIC-14 (shown as black symbols). Day 1 sera from two dogs (ITIC-10 and ITIC-15) inhibited binding. A single serum sample, ITIC-14 Day 30, showed a significant CAHA response but did not inhibit binding (shown as the black circle).

Significant CAHA at various timepoints was detected by ELISA in 7 of 12 dogs: i) ITIC-06 and ITIC-09 from Arm A at Day 30; ii) ITIC-08 from Arm B at Day 60; iii) ITIC-14 from Arm B at Day 30; and iv) ITIC-05, ITIC-11, and ITIC-15 from Arm B at Day 30 and Day 60 (Fig 3). Except for the Day 30 sera from ITIC-14, the above sera also significantly inhibited 14G2a from binding to GD2 in the flow cytometry assay (Fig 4). To evaluate the relationship between the ELISA and binding inhibition, the values for each serum sample for each subject are plotted in Fig 5, with the ELISA O.D. value plotted on the Y axis, and the % binding inhibition value plotted on the X-axis. There were no significant correlations between the two assays within or for both arms combined detected. Significant binding inhibition was detected earlier than significant CAHA: i) at Day 1 in 2 dogs; and ii) at Day 10 in 10 dogs. Overall, inhibition was detected in 10 of 12 dogs: i) 5 of 6 Arm A dogs and 5 of 6 Arm B dogs at Day 10; ii) 5 of 6 Arm A dogs and 5 of 6 Arm B dogs at Day 30; iii) 3 of 4 Arm A dogs and 5 of 6 Arm B dogs at Day 60. Sera from the two dogs that did not inhibit binding in the flow cytometry assay (ITIC-13 and ITIC-14) were also CAHA negative by ELISA except for the Day 30 sera from ITIC-14 (Figs 3 and 4). Healthy canine sera at the same dilution as sera from the clinical trial dogs were negative for CAHA and did not inhibit the binding of 14G2a to M14 (S8 Fig).

## Discussion

We developed two distinct assays to monitor and assess the CAHA response in dogs receiving treatment of a readily palpable oral malignant melanoma with local RT plus IT injection of a humanized IC. An ELISA was designed to detect CAHAs specifically targeting human IgG – which is analogous to the Fc portion of hu14.18-IL2 [31]. We also utilized a flow cytometry assay to investigate the inhibition of anti-GD2 mAb binding to GD2 by sera from dogs receiving RT and IT-IC. This approach employed ELISA as a tool to identify the presence of ADA in serum, followed by a subsequent flow cytometry analysis to provide additional characterization of the ADA response.

Although there are commercially available assays to detect various anti-species antibodies, e.g., mouse anti-human (MAHA) and human anti-mouse (HAMA), an off-the-shelf assay is not available to detect CAHA. The absence of a commercially available ELISA specifically designed to detect CAHAs is an unmet need within the comparative immuno-oncology field. This gap becomes increasingly relevant as the canine model gains recognition for its value in studying human disease. The primary objective of developing our in-house CAHA ELISA was to create a monitoring tool that would be easy to use in veterinary and translational research laboratories. Additionally, we sought to develop a cost-effective and flexible assay. The binding specificity of the CAHA could also be explored in more depth by modifying the whole human IgG coating reagent that was used in this report (e.g., whole IgG, Fc fragment, Fab or F(ab’)_2_).

A challenge in developing an ELISA to detect canine antibody that binds human IgG was the lack of commercially available anti-canine IgG HRP conjugates that were cross adsorbed against human IgG. This cross-adsorption step is crucial for reducing the assay background and ensuring specificity. To address this limitation, we used a rabbit anti-mouse IgG HRP conjugate that had been cross adsorbed against human IgG. In addition, we included a commercially available mouse IgG specific for human IgG as a positive control analyte sample for detection by the rabbit anti-mouse HRP in each assay. The selection of antibodies and the incorporation of cross-adsorption steps contributed to the assay’s specificity and adaptability for potential use in both veterinary and translational research lab settings.

Although primary-only and secondary-only control wells were not included in the final ELISA, nonspecific background was addressed during assay development. An in-house adsorption of the rabbit anti-dog IgG HRP secondary reagent with plate-bound human IgG failed to sufficiently reduce assay background. Thus, the anti-dog IgG HRP was replaced with a commercially cross-adsorbed rabbit anti-mouse IgG HRP reagent, which effectively minimized background in PBS-only wells, i.e., wells coated with human IgG but without the mouse anti-human IgG positive control. The mouse anti-human IgG monoclonal antibody was used as in internal control during the assay to confirm HRP activity and effective signal development but was not used to generate a standard curve. This positive control was therefore interpreted qualitatively, based on visible color change, rather than correlation with known antibody concentrations.

The Negative Control wells were included on each plate as assay controls. We did not perform formal statistical analyses of the Negative Control to any canine sera nor to the mouse anti-human IgG positive control. The lack of a substantial difference between the negative control wells with PBS and the pre-treatment samples suggests the lack of interfering substances in canine sera. Not only were all sera from a dog assayed on the same plate but also sera from multiple dogs were assayed concurrently on the same plate. Moreover, to be fiscally responsible, the Negative Control wells were run as one set of quadruplicate wells per plate and not per dog or per sera sample. Sera from not only ITIC-11 and ITIC-12, but also ITIC-07, were assayed on the same day and on the same plate explaining the similarity in the Negative Control OD values for these three dogs. That the Negative Control OD values for these three dogs was higher than the other 9 dogs is likely due to day-to-day assay variability and is further support for not comparing individual dogs to one another.

It is important to note that while our in-house ELISA assay is suitable for the exploratory detection of CAHA in translation research context, they do not meet the sensitivity or validation standards required for regulatory grade ADA assets, as set forth by the 2019 FDA guidance on assay development [66]. Additionally, the FDA guidelines highlight a recommendation to account for pre-existing antibodies during ADA analysis. The early binding inhibition seen in two dogs prior to IC administration may reflect such pre-existing cross-reactive antibodies.

Our CAHA ELISA is primarily designed to detect free ADAs in serum by detecting their binding to plate-bound human IgG. In contrast, the in vitro binding inhibition flow cytometry assay allows the ADAs to interact with the soluble mouse 14.G2a mAb (which shares the idiotype of the hu14.18-IL2 IC) in solution, and evaluates detection of GD2 binding in a cellular context, enabling the detection of ADAs that inhibit or interfere with the binding of a therapeutic drug to its specific target in its natural form on the cell surface, e.g., GD2 on tumor cells. This distinction is critical because antigens immobilized to plastic, as in an ELISA, may not accurately mimic the natural conformations of antigens on cell surfaces [67], potentially leading to an underestimation of certain ADAs by this ELISA that flow cytometry can detect.

It is of interest that sera from two dogs (ITIC-10 and ITIC-15) inhibited the binding of the anti-GD2 mAb to GD2 on target cells at Day 1. The Day 1 sample was collected the day of, but prior to, the first injection of hu14.18-IL2. Therefore, these responses following RT may reflect pre-existing canine anti-human or anti-mouse antibodies, cross species-reactive antibodies, or assay interference [68–70]. The first step in the flow cytometry binding inhibition assay is the incubation of canine sera with the mouse anti-GD2 mAb 14G2a prior to incubating the mixture with the GD2 + M14 cells. Therefore, a possible explanation for the Day 1 binding inhibition is pre-existing canine anti-mouse antibodies as previously reported [69], but these would have needed to appear, or be boosted, following baseline, as the positive binding inhibition is detected in comparison to the baseline value for that same dog. We did not prospectively survey clients regarding their pet’s known allergies or exposures to other mammalian species, e.g., mouse or rabbit, which could potentially induce a humoral response. mAbs of both mouse and rabbit origin are used in our in-house assays and could result in assay background or interference. However, neither of these two dogs were CAHA+ by ELISA at Day 1. Anti-human IgG was not detected by ELISA until Day 30 in any dog, nor from healthy canine sera, suggesting exposure to hu14.18-IL2 as the origin of the antibodies. However, we have not formally tested for pre-existing anti-mouse or anti-rabbit antibodies in these dogs. These seemingly discordant data could reflect the different assay sensitivities and different assay modalities. Moreover, serum from healthy humans and healthy dogs can both contain detectable levels of autoantibodies including rheumatoid factors and anti-idiotypic antibodies which may cause assay interference [68,70,71].

Additionally, activation of B cells, generation of anti-tumor antibodies and establishment of B cell memory following a treatment regimen similar to that used in this trial have been reported in a mouse melanoma model [72]. As IgM typically arises early in an immune response, the CAHA response detected at early timepoints (e.g., Day 1, Day 10) in this study may be of IgM isotype which our CAHA ELISA may also detect. in contrast, the flow cytometry assay does not detect a particular antibody isotype and thus may detect ADA despite lack of CAHA detected by ELISA in the same serum. The detection of binding inhibition at early time points may also result from the isotype agnostic nature of the flow cytometry assay. The increase in binding inhibition observed in two dogs (ITIC-10 and ITIC-15) from baseline to Day 1 was unexpected as the dogs had not been exposed to hu14.18-IL2 at the time of serum collection on either day. Moreover, the timeframe from baseline to Day 1 in this study was between 5–9 days, representing rapid kinetics for a *de novo* humoral response. However, we have not determined the fine specificity of the antibody responses in this study. All dogs treated in this study received radiation, thus there is potential for radiation-induced antigen shedding by tumor to trigger a humoral response [73]. Tumor-specific and self-specific antibodies have been found in sera of cancer patients in several studies [74,75]. Thus, the binding inhibition at Day 1 in these two dogs could be due to endogenous cross-reactive antibodies or those recognizing cryptic epitopes or neoantigens [76].

The IC has humanized IL2 linked to the mAb Fc region, thus the IL2 and/or the linker between the Fc and the IL2 may be recognized as foreign in the dog triggering an immune response. None of the dogs in this study experienced significant or unexpected adverse events [35]. However, as dogs received only a single 3-day course of IT-IC, we could not evaluate the impact of induced ADAs on subsequent pharmacokinetics of hu14.18-IL2 or on the potential toxicity during subsequent courses. It will be important to evaluate peak serum levels of hu14.18-IL2 as well as monitor anti-IL2 and anti-linker responses to determine if CAHA+ sera affect antibody-mediated cellular cytotoxicity, as seen in some humans receiving IC treatment [44].

Additional studies could further characterize the binding specificity in terms of distinct IgG regions, e.g., Fc receptor, Fab hinge region [69]. Further, as in humans, IgG is the most prevalent Ig in canine sera, however IgM and IgA are present at lower concentrations [77]. The rabbit anti-mouse IgG HRP secondary reagent used in the ELISA is reactive to both the heavy and light chain of mouse IgG. As such, this reagent may also react with other immunoglobulin classes, e.g., IgM, IgA, since these all share the same light chain. Therefore, subsequent testing could determine the isotype of the CAHA responses, as well as species cross-reactivity – in particular to mouse or rabbit IgG.

The timeline of the anti-human IgG CAHA ELISA response observed in our study (7 of 12 dogs at Day 30) is similar to that observed in a previous study of dogs treated with NHS-IL12, a humanized antibody against necrotic tumor regions linked to humanized IL12. Dogs treated with NHS-IL12 developed ADA against both human IgG (8 of 12 dogs) and against human IL12 (5 of 14 dogs) between 15–29 days after treatment [51]. Factors that may explain differences in the ADA timeline between our study and that of Paoloni et al., include the composition and target of the IC, IC dose level, administration route, and the assays used to monitor the development of ADA.

## Conclusion

The CAHA detection assays in this report provide tools to monitor parallel canine patient populations receiving treatment with human biopharmaceuticals. These assays should be translatable to other treatment regimens delivering “foreign” protein molecules *in vivo*, as well as to species other than the dog, provided appropriate controls are available. The recent availability of immune checkpoint blockade in the form of a caninized anti-PD1 therapeutic mAb (Gilvetmab, Merck) expands the treatment options for dogs with melanoma. To this end, we are evaluating the addition of immune checkpoint blockade, i.e., anti-PD1, to our novel treatment regimen of RT and IT-IC with hu14.18-IL2 in dogs with melanoma. Local anti-tumor responses as well as systemic immune responses, including the development of CAHA using the assays described herein, will be investigated.

## Supporting information

S1 Fig Study flowchart. Number of patients screened and randomized to receive treatment and number of samples assayed for CAHA. Created in BioRender. Zuleger, C. (2025) https://BioRender.com/3c9ma17
(TIF)

S2 Fig Cell line authentication. The GD2 positive melanoma cell line M14 used in this study matched the STR profile of the human cell line M14 (CVCL_1395) with 24/26 matching alleles (92.3%) and is thus a called a match to M14 (CVCL_1395).
(TIF)

S3 Fig Gating strategy for GD2 + M14 cell. A series of gates are applied to: (A) exclude events collected during unstable flow by plotting Time vs. scatter, (B) exclude forward scatter doublets, (C) exclude side scatter doublets, and (D) gate on cells of interest. (E) GD2 PE fluorescence signal is visualized in a histogram. Shown is M14 incubated in the presence of Day 1 sera from ITIC-15.
(TIF)

S4 Fig Rabbit anti-dog IgG HRP results in high background. Rabbit anti-dog IgG HRP yields high background when used as the secondary reagent in the ELISA. The absorbance read-out does not correlate with the concentration of the positive control mouse anti-human IgG mAb (compare absorbance across rows). Further, a substantial signal was detected in the absence of the positive control mouse anti-human IgG mAb (note absorbances in column 6). Created in https://BioRender.com
(TIF)

S5 Fig Cross-adsorption of rabbit anti-dog IgG HRP does not reduce background to acceptable levels. Cross-adsorbing the rabbit anti-dog IgG HRP with excess human IgG did not substantially reduce the high background. The absorbance of wells developed with rabbit anti-dog IgG HRP that was not cross-adsorbed (rows A-C) was similar to those developed with our in-house cross-adsorbed rabbit anti-dog IgG HRP (rows E-G). The background was high even in the absence of the mouse anti-human IgG positive control, e.g., columns 3, 6, and 9 suggesting that the rabbit anti-dog IgG may bind directly to the human IgG coated wells. Created in https://BioRender.com
(TIF)

S6 Fig Cross-adsorbed rabbit anti-mouse IgG HRP greatly reduces background. A commercially available, rabbit anti-mouse IgG HRP cross-adsorbed against human serum proteins greatly reduced the non-specific background to acceptable levels while the positive control murine anti -human IgG signa was preserved. Created in https://BioRender.com
(TIF)

S7 Fig CAHA is induced in some dogs receiving IT-IC injections. Data represented are the individual replicate CAHA values, and the respective means and standard deviations from each dog. The data are those that generated the summary Fig 3. CAHA levels measured as O.D. values by ELISA, from sera assayed in quadruplicate collected at Baseline (before radiation), Day 1 (after radiation, but prior to IT-IC), and Days 10, 30 and 60 (after IT-IC) with standard deviation error bars are shown. Day 60 timepoint was not available for two dogs, ITIC-06 and ITIC-12, in Arm A. Increases in absorbance from Baseline to Day 1, Day 10, Day 30, or Day 60 for each dog were analyzed and significance indicated as * p < 0.05; ** p < 0.01; *** p < 0.001.
(TIF)

S8 Fig Binding inhibition assay controls. PE-conjugated anti-GD2 antibody 14G2a was mixed with either (A) 20 μl (open histogram with solid line) or (B) 2.5 μl (open histogram with solid line) healthy canine sera before staining M14 cells. M14 stained with 14G2a-PE mixed with 20 μl or 2.5 μl PBS as a no-serum control is represented as a gray histogram in both (A) and (B). Data are representative of triplicates. Values represent % binding inhibition compared to no-serum control.
(TIF)

S9 Fig Binding of anti-GD2 antibody to GD2 target in the presence of canine sera. Data represented are the individual replicate values, and the respective means and standard deviations from each dog. The Median Fluorescence Intensity (MFI) data are those used to calculate the % binding inhibition presented in the summary Fig 4. Data shown are triplicates as described in Materials and Methods. Sera was collected at Baseline (before radiation), Day 1 (after radiation, but just prior to IT-IC), and Days 10, 30 and 60 (after IT-IC). Day 60 timepoint was not available for two dogs, ITIC-06 and ITIC-12, in Arm A. Significance indicated is based on mean of triplicate MFI values at Baseline compared to post-treatment timepoints, * p < 0.05; ** p < 0.01; *** p < 0.001.
(TIF)

S10 Fig Serum from dogs treated with hu14.18-IL2 shows varied level of inhibition of 14G2a binding to target GD2. Prior to staining M14 cells, PE-conjugated anti-GD2 antibody 14G2a was mixed with baseline/pre-treatment sera or post-treatment sera from different timepoints from (A) ITIC-14 or (B) ITIC-15. Baseline/pre-treatment data are represented as gray histograms; post-treatment data are represented as open histograms with solid lines. Data are representative of triplicates. Values represent % binding inhibition compared to baseline/pre-treatment control.
(TIF)
