# Supplementary figures and images for: In vitro detection of canine anti-human antibodies following intratumoral injection of the hu14.18-IL2 immunocytokine in spontaneous canine melanoma
Source: PLoS One. 2025 Aug 19;20(8):e0330200. doi: 10.1371/journal.pone.0330200 (PMC12364362; doi:10.1371/journal.pone.0330200)

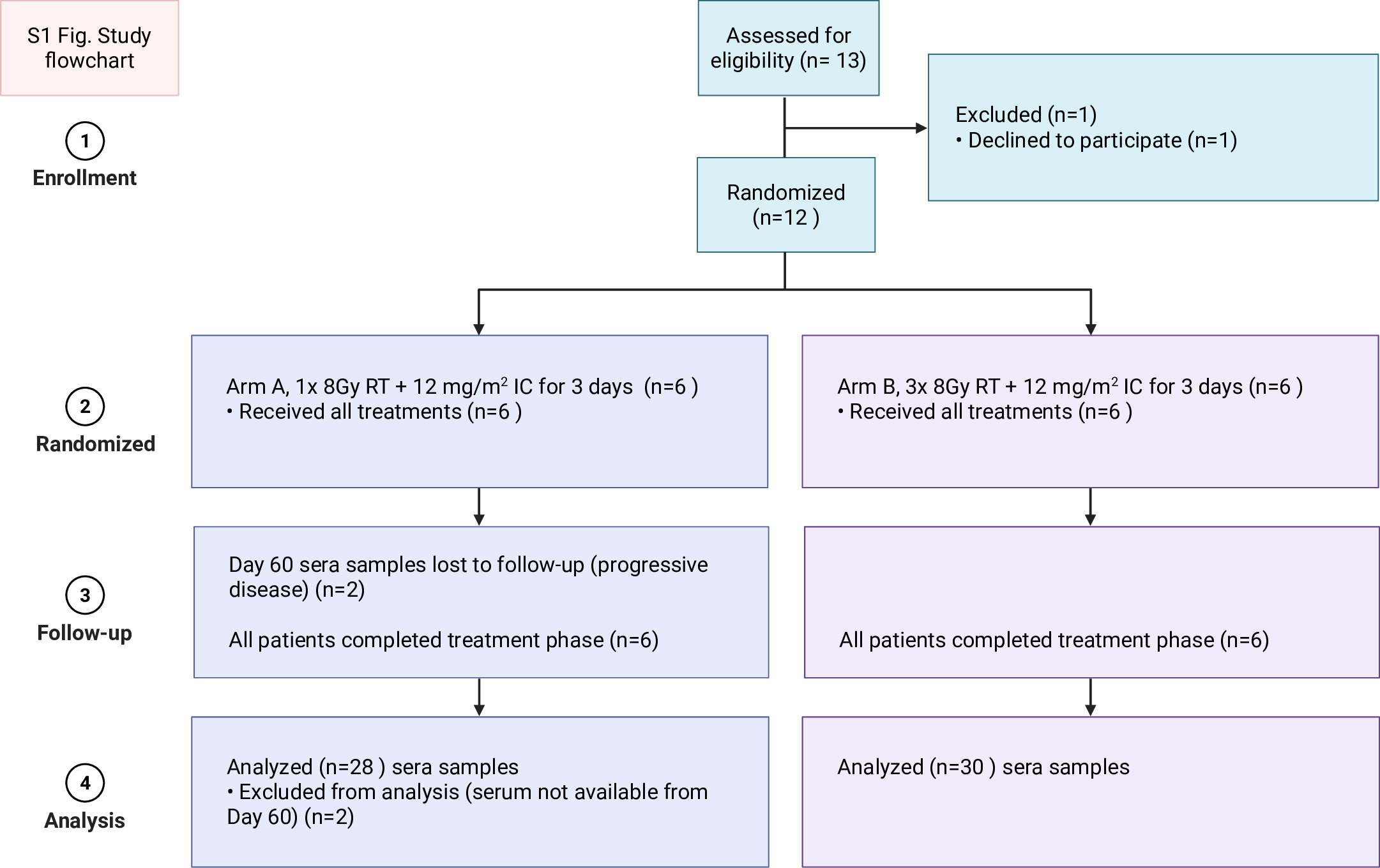

Supplement: S1 Fig — Number of patients screened and randomized to receive treatment and number of samples assayed for CAHA. Created in BioRender. Zuleger, C. (2025) https://BioRender.com/3c9ma17 (TIF) [file pone.0330200.s001.tif]

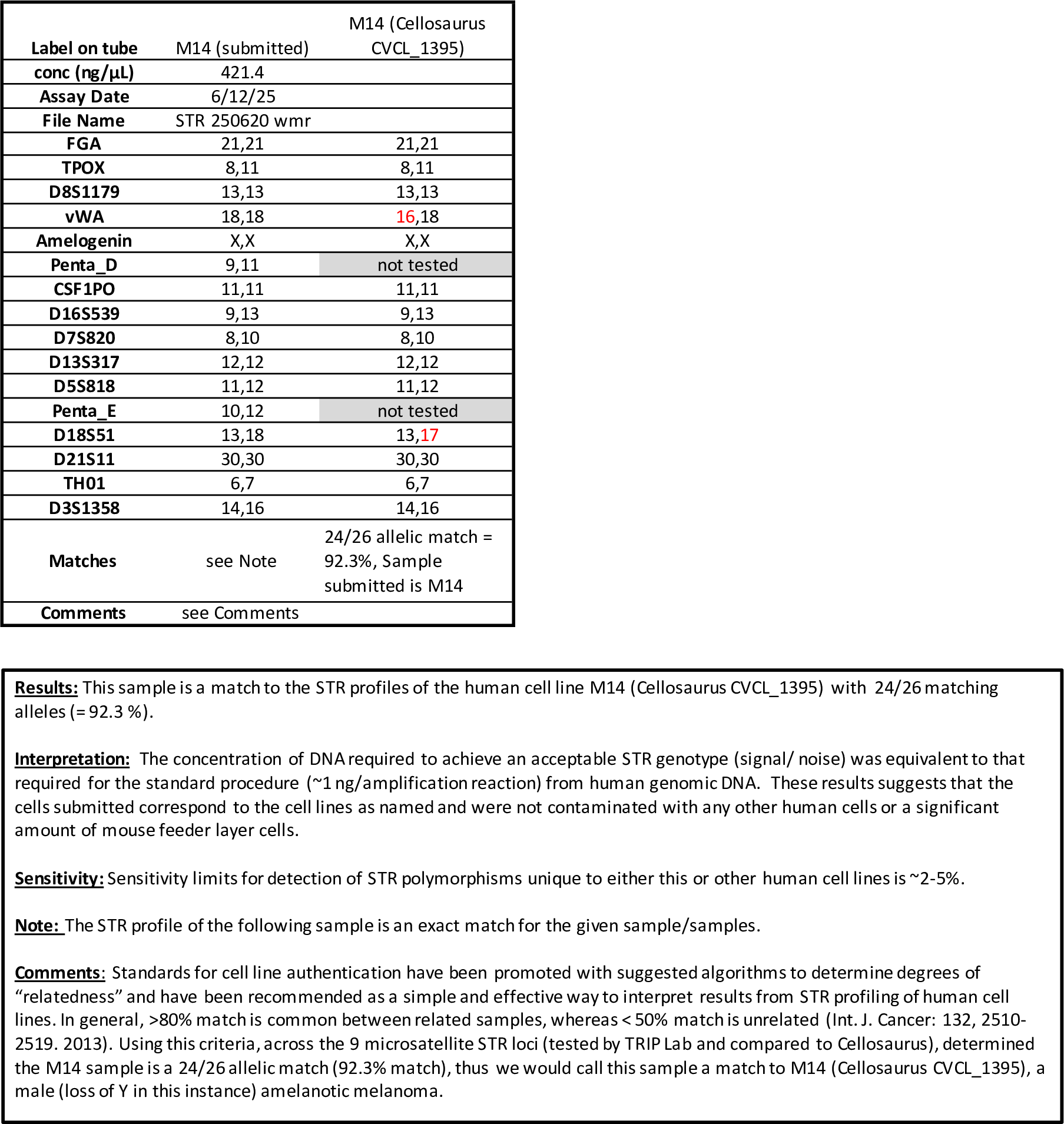

Supplement: S2 Fig — The GD2 positive melanoma cell line M14 used in this study matched the STR profile of the human cell line M14 (CVCL_1395) with 24/26 matching alleles (92.3%) and is thus a called a match to M14 (CVCL_1395). (TIF) [file pone.0330200.s002.tif]

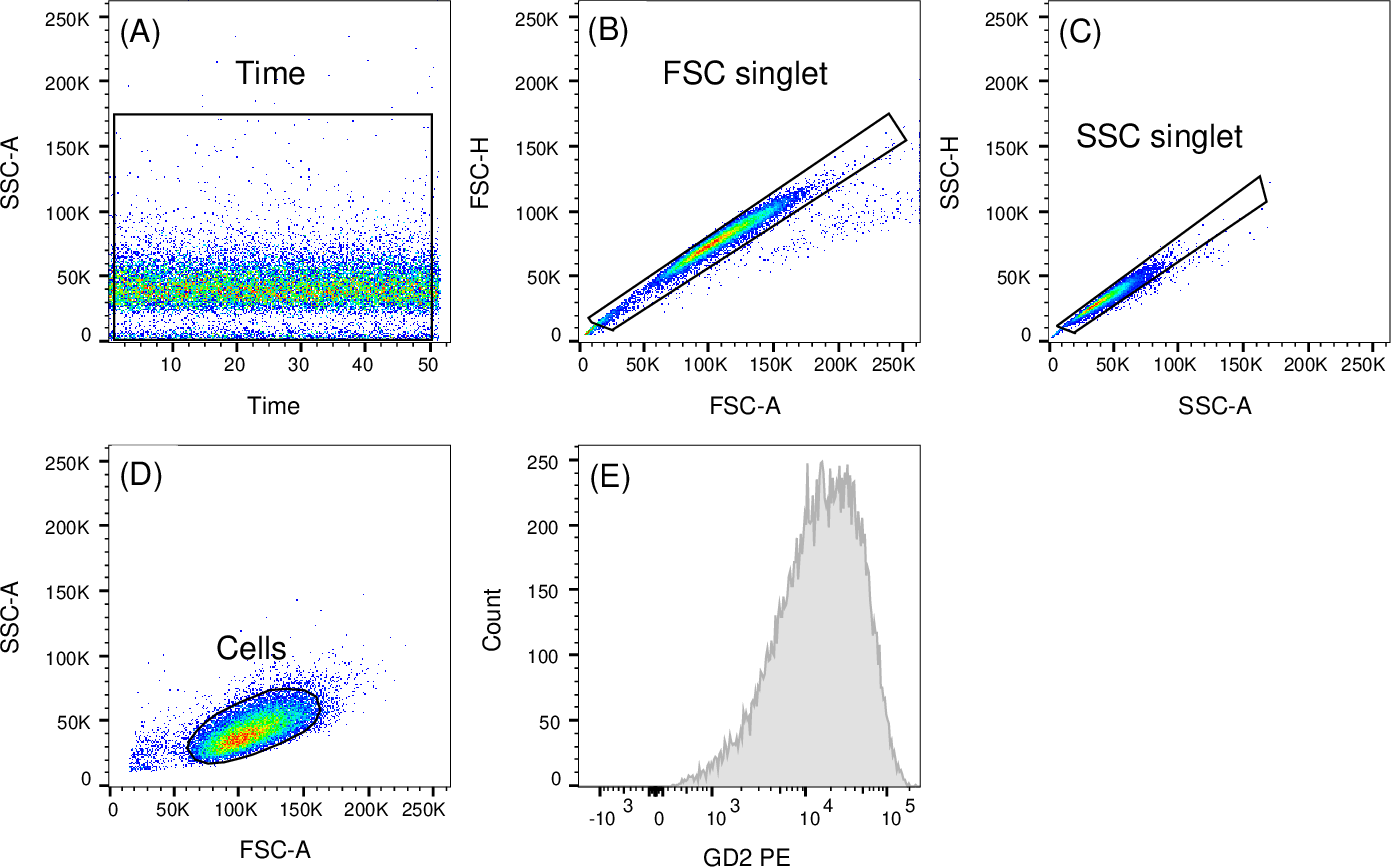

Supplement: S3 Fig — A series of gates are applied to: (A) exclude events collected during unstable flow by plotting Time vs. scatter, (B) exclude forward scatter doublets, (C) exclude side scatter doublets, and (D) gate on cells of interest. (E) GD2 PE fluorescence signal is visualized in a histogram. Shown is M14 incubated in the presence of Day 1 sera from ITIC-15. (TIF) [file pone.0330200.s003.tif]

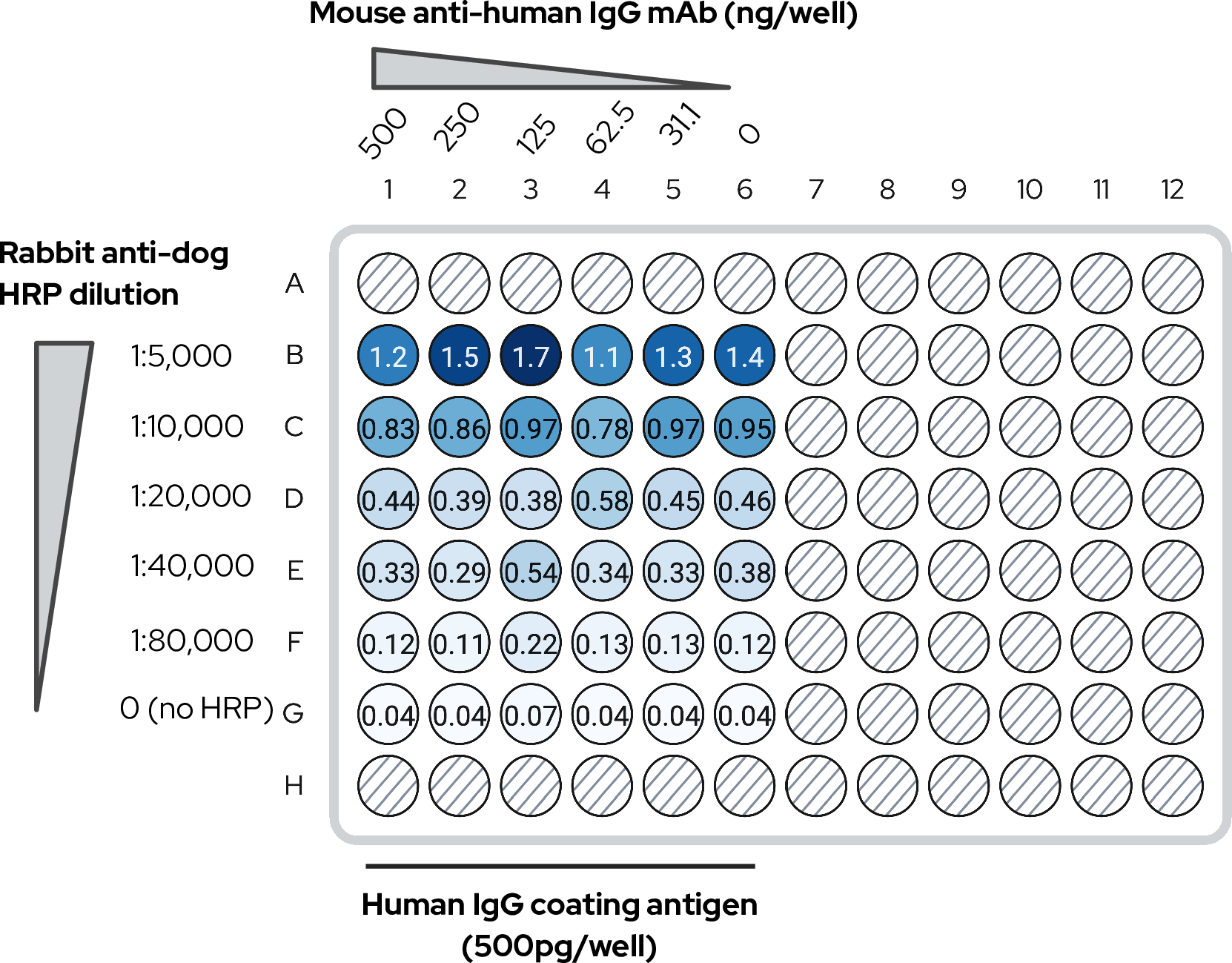

Supplement: S4 Fig — Rabbit anti-dog IgG HRP yields high background when used as the secondary reagent in the ELISA. The absorbance read-out does not correlate with the concentration of the positive control mouse anti-human IgG mAb (compare absorbance across rows). Further, a substantial signal was detected in the absence of the positive control mouse anti-human IgG mAb (note absorbances in column 6). Created in https://BioRender.com (TIF) [file pone.0330200.s004.tif]

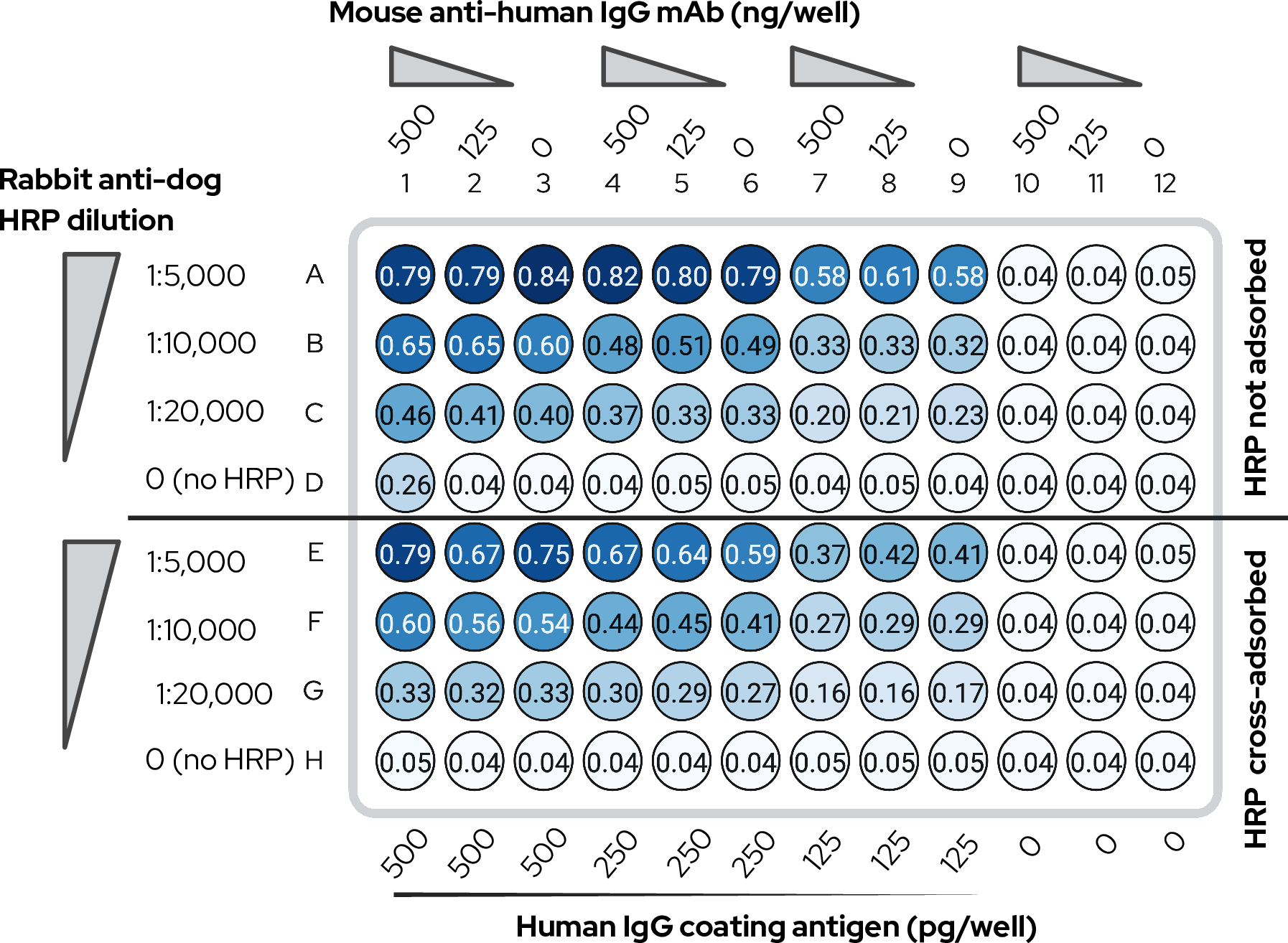

Supplement: S5 Fig — Cross-adsorbing the rabbit anti-dog IgG HRP with excess human IgG did not substantially reduce the high background. The absorbance of wells developed with rabbit anti-dog IgG HRP that was not cross-adsorbed (rows A-C) was similar to those developed with our in-house cross-adsorbed rabbit anti-dog IgG HRP (rows E-G). The background was high even in the absence of the mouse anti-human IgG positive control, e.g., columns 3, 6, and 9 suggesting that the rabbit anti-dog IgG may bind directly to the human IgG coated wells. Created in https://BioRender.com (TIF) [file pone.0330200.s005.tif]

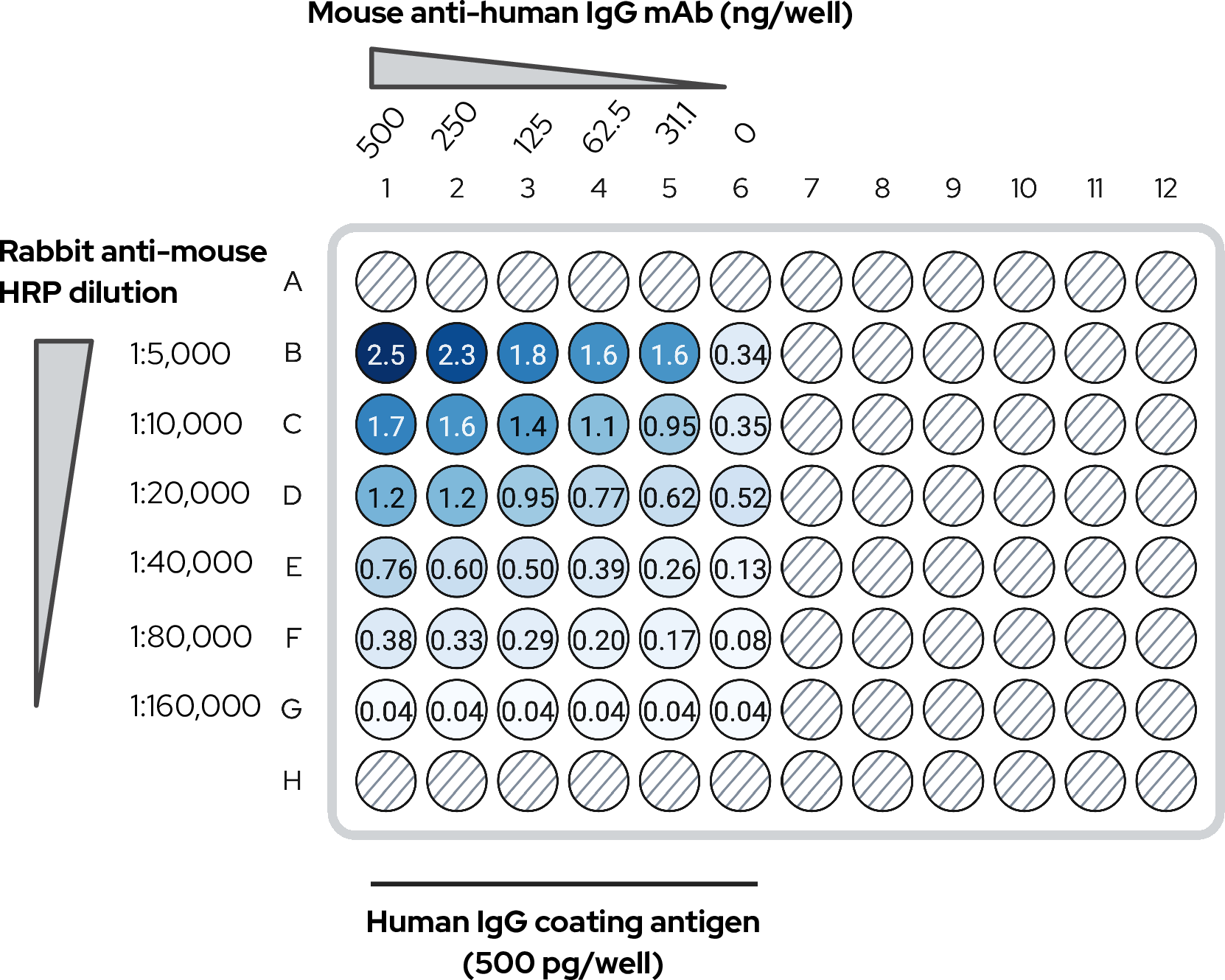

Supplement: S6 Fig — A commercially available, rabbit anti-mouse IgG HRP cross-adsorbed against human serum proteins greatly reduced the non-specific background to acceptable levels while the positive control murine anti -human IgG signa was preserved. Created in https://BioRender.com (TIF) [file pone.0330200.s006.tif]

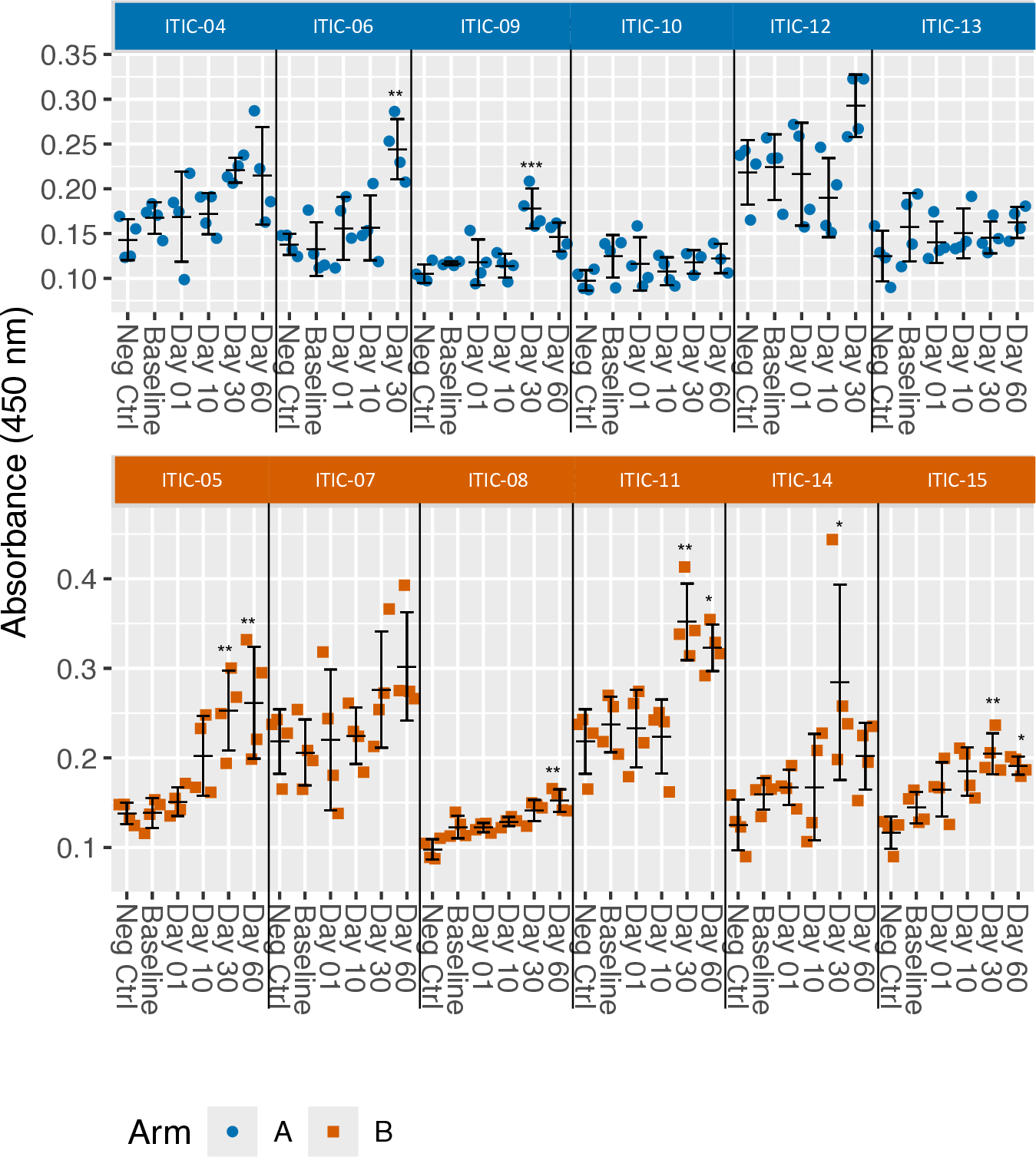

Supplement: S7 Fig — Data represented are the individual replicate CAHA values, and the respective means and standard deviations from each dog. The data are those that generated the summary Fig 3. CAHA levels measured as O.D. values by ELISA, from sera assayed in quadruplicate collected at Baseline (before radiation), Day 1 (after radiation, but prior to IT-IC), and Days 10, 30 and 60 (after IT-IC) with standard deviation error bars are shown. Day 60 timepoint was not available for two dogs, ITIC-06 and ITIC-12, in Arm A. Increases in absorbance from Baseline to Day 1, Day 10, Day 30, or Day 60 for each dog were analyzed and significance indicated as * p < 0.05; ** p < 0.01; *** p < 0.001. (TIF) [file pone.0330200.s007.tif]

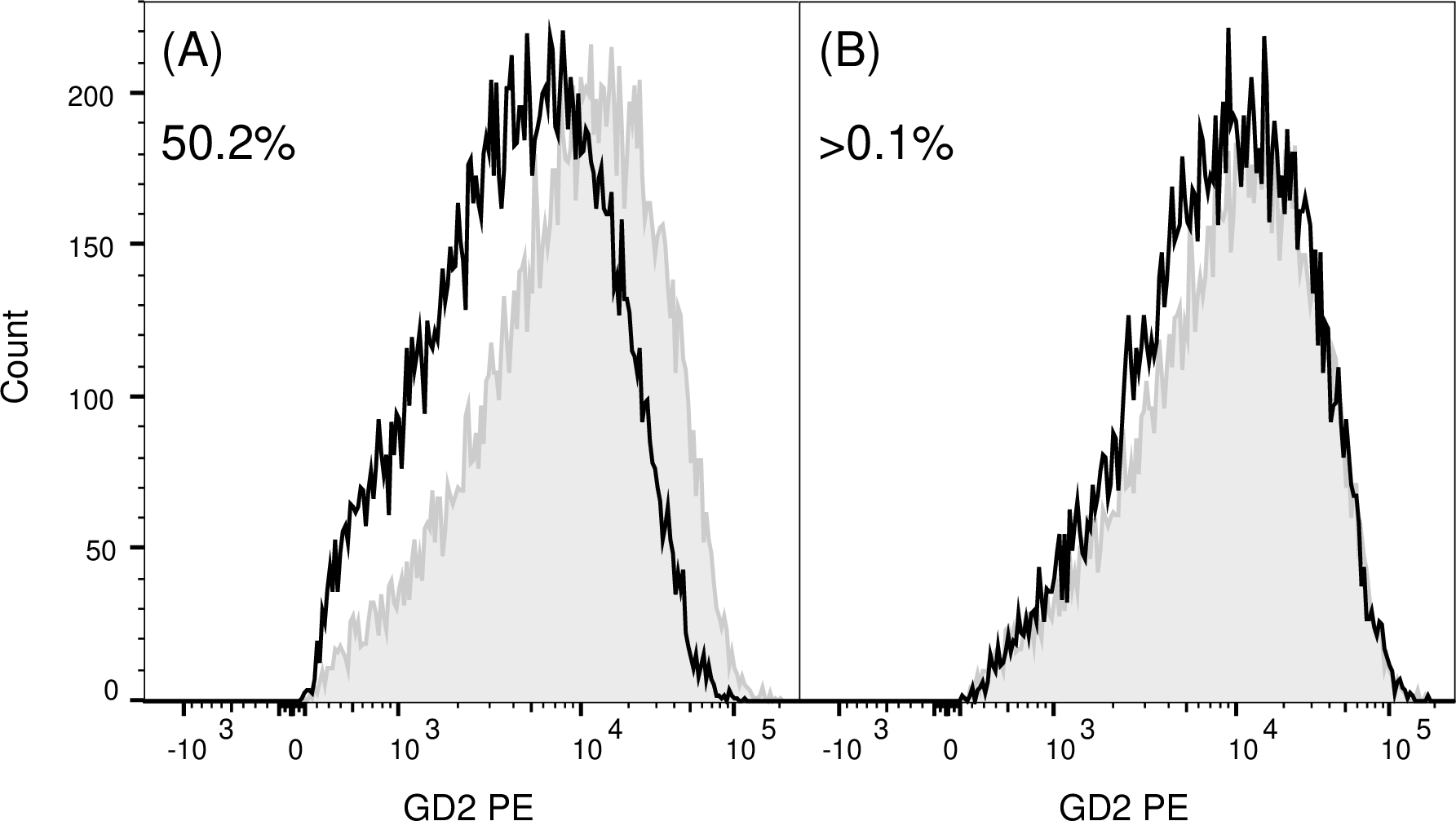

Supplement: S8 Fig — PE-conjugated anti-GD2 antibody 14G2a was mixed with either (A) 20 μl (open histogram with solid line) or (B) 2.5 μl (open histogram with solid line) healthy canine sera before staining M14 cells. M14 stained with 14G2a-PE mixed with 20 μl or 2.5 μl PBS as a no-serum control is represented as a gray histogram in both (A) and (B). Data are representative of triplicates. Values represent % binding inhibition compared to no-serum control. (TIF) [file pone.0330200.s008.tif]

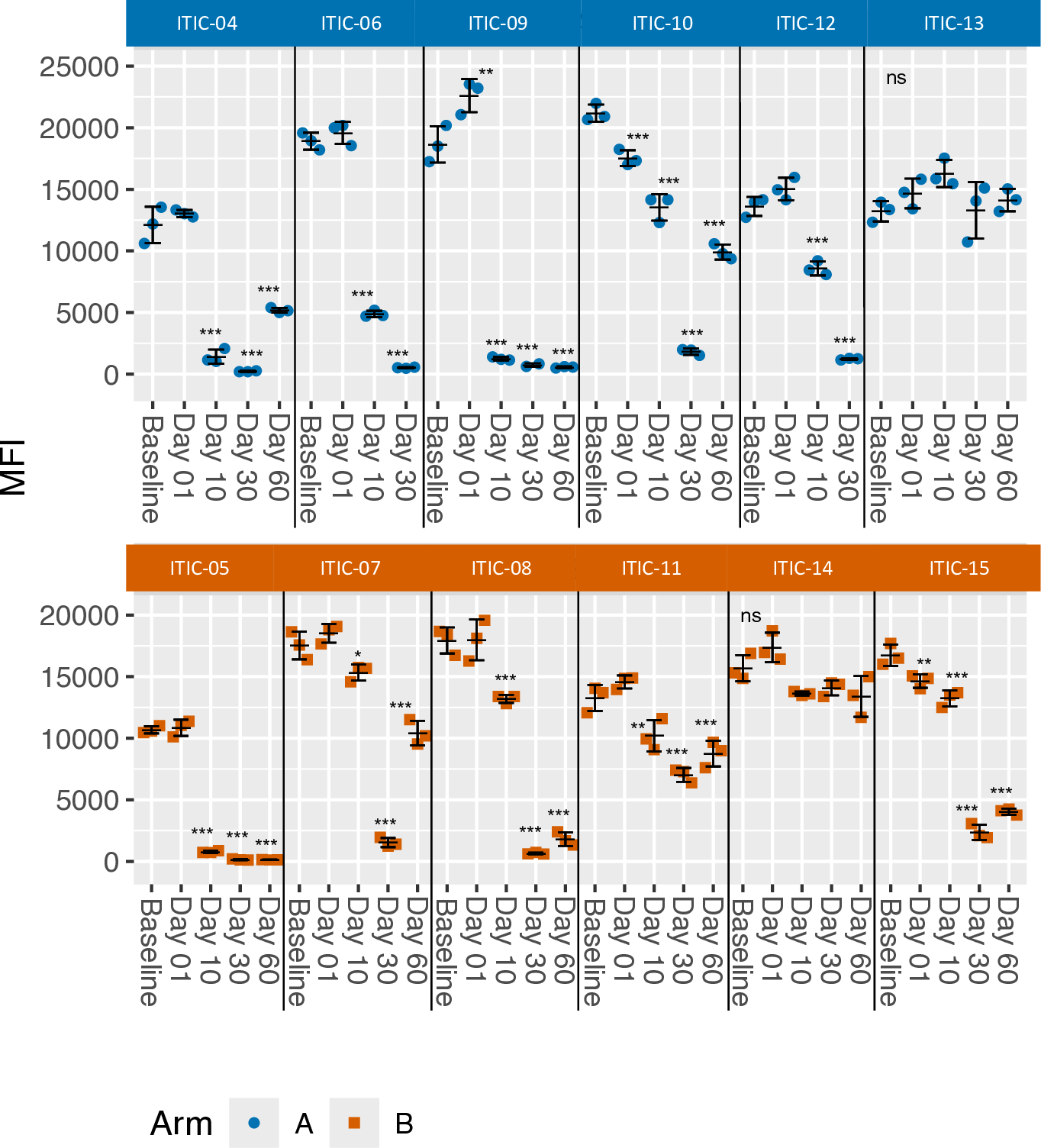

Supplement: S9 Fig — Data represented are the individual replicate values, and the respective means and standard deviations from each dog. The Median Fluorescence Intensity (MFI) data are those used to calculate the % binding inhibition presented in the summary Fig 4. Data shown are triplicates as described in Materials and Methods. Sera was collected at Baseline (before radiation), Day 1 (after radiation, but just prior to IT-IC), and Days 10, 30 and 60 (after IT-IC). Day 60 timepoint was not available for two dogs, ITIC-06 and ITIC-12, in Arm A. Significance indicated is based on mean of triplicate MFI values at Baseline compared to post-treatment timepoints, * p < 0.05; ** p < 0.01; *** p < 0.001. (TIF) [file pone.0330200.s009.tif]

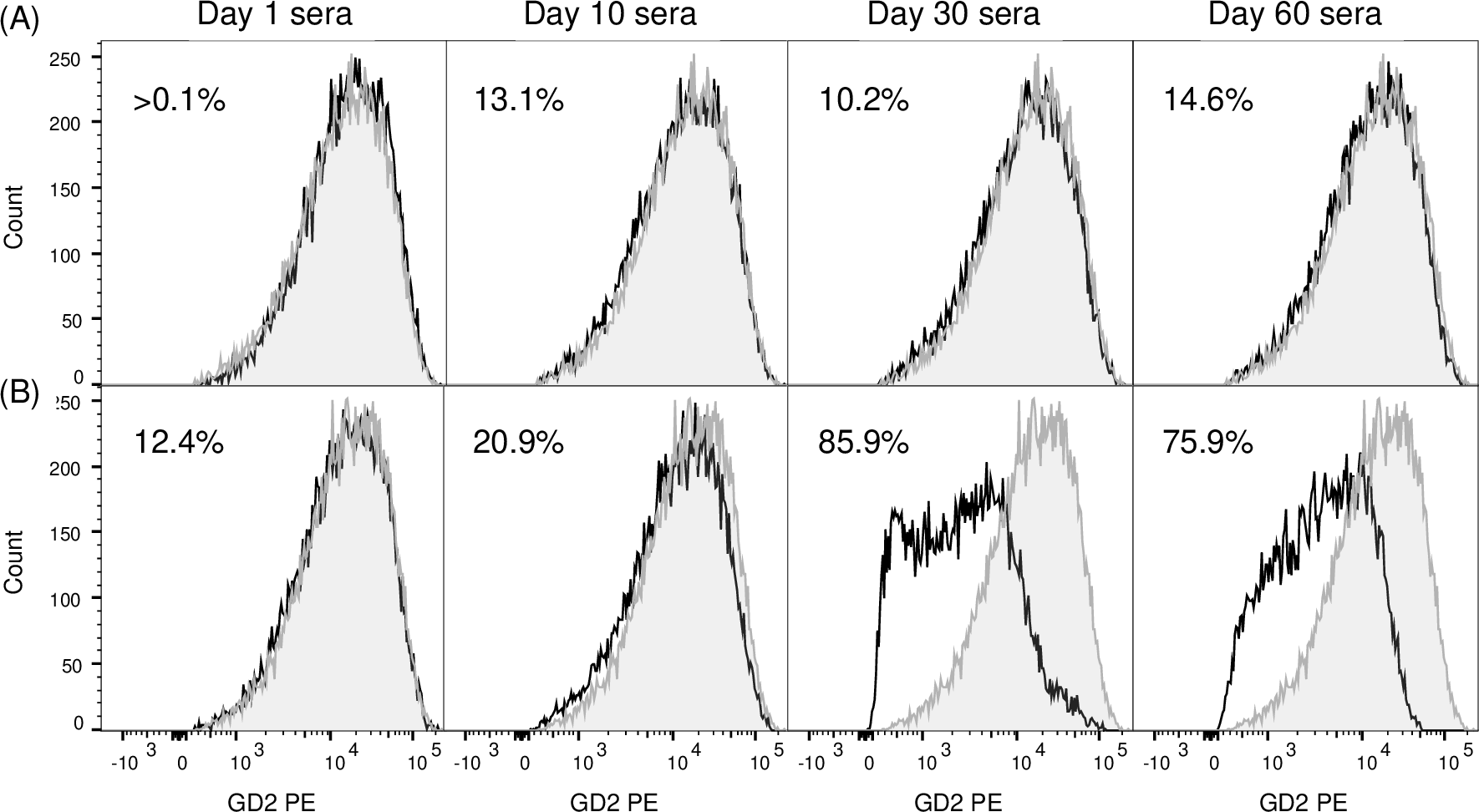

Supplement: S10 Fig — Prior to staining M14 cells, PE-conjugated anti-GD2 antibody 14G2a was mixed with baseline/pre-treatment sera or post-treatment sera from different timepoints from (A) ITIC-14 or (B) ITIC-15. Baseline/pre-treatment data are represented as gray histograms; post-treatment data are represented as open histograms with solid lines. Data are representative of triplicates. Values represent % binding inhibition compared to baseline/pre-treatment control. (TIF) [file pone.0330200.s010.tif]
